# Multi-Platform Omics Analysis for Identification of Molecular Characteristics and Therapeutic Targets of Uveal Melanoma

**DOI:** 10.1038/s41598-019-55513-z

**Published:** 2019-12-17

**Authors:** Yong Joon Kim, Seo Jin Park, Kyung Joo Maeng, Sung Chul Lee, Christopher Seungkyu Lee

**Affiliations:** 1 Department of Ophthalmology, Institute of Vision Research, Severance Hospital, Yonsei University College of Medicine, Seoul, Republic of Korea; 2 Department of Ophthalmology, Institute of Vision Research, Gangnam Severance Hospital, Yonsei University College of Medicine, Seoul, Republic of Korea

**Keywords:** Eye cancer, Oncogenesis

## Abstract

Currently, there is no effective treatment for metastatic uveal melanoma (UVM). Here, we aimed to identify the mechanism involving intrinsic chemoresistance of metastatic UVM and the relevant therapeutic targets for UVM. We analyzed cohorts of 80 and 67 patients with primary UVM and skin cutaneous melanoma (SKCM), respectively, using The Cancer Genome Atlas dataset. Mutational burdens identified by whole exome sequencing were significantly lower in UVM than in SKCM patients. COSMIC mutational signature analysis identified that most of the mutations in UVM patients (>90%) were associated with spontaneous deamination of 5-methylcytosine or defective mismatch repair. Transcriptome analysis revealed that the MYC signature was more enriched in UVM patients, as compared to SKCM patients. Fifty-nine (73.8%) of 80 UVM patients showed gains in MYC copy number, and a high MYC copy number was associated with aggressive clinicopathological features of tumors and poor survival. Kinome-wide siRNA library screening identified several therapeutic targets, reported as synthetic lethal targets for MYC-addicted cancers. Notably, UVM cell lines showed high susceptibility to a WEE1 inhibitor (MK-1775; adavosertib) at a clinically tolerable dose. Overall, our study identified high MYC activity in UVM, and suggested G2/M checkpoint inhibitors as effective therapeutic targets for UVM.

## Introduction

Uveal melanoma (UVM) and skin cutaneous melanoma (SKCM) are both derived from melanocytes but show different characteristics, including significant differences in tumorigenesis, genetic alterations, and therapeutic responses^1^. The BRAF V600E mutation commonly seen in SKCM patients is not frequently observed in UVM patients, and GNAQ/GNA11, SF3B1, EIF1AX, and BAP1 mutations seen in UVM patients are rarely observed in SKCM patients^2,3^. Vemurafenib, a targeted agent for BRAF V600E, significantly improves therapeutic outcomes in SKCM patients^4^. The GNAQ/GNA11 mutations frequently observed in UVM patients have also been reported to activate the MAPK pathway, but clinical trials with the MEK1/2 inhibitor, selumetinib, have failed to improve survival^5–7^.

The difference in therapeutic outcomes between the two tumors has been further verified by the recent introduction of immune checkpoint inhibitors. Immunotherapy has led to favorable therapeutic outcomes for SKCM patients^8^. Some studies have reported the response to immune checkpoint inhibitors in UVM, but the effect was limited to minority of UVM^9–12^. Despite excellent local control, blood-borne distant metastasis occurs in up to 50% of UVM patients and approximately 85% of patients with metastasis die within 1 year^13^. Currently, there is no effective treatment for metastatic UVM, and the mechanism involving chemoresistance to most treatments remains poorly understood. In this study, we analyzed the whole exome sequencing and transcriptome (mRNA) sequencing data of UVMs and SKCMs in The Cancer Genome Atlas (TCGA) dataset, and performed kinome-wide siRNA library screening to characterize UVM and to identify potential therapeutic targets of UVM.

## Results

### Study population

Eighty and 67 patients with primary UVMs and SKCMs, respectively, in TCGA datasets were identified and included in this study. The mean ages of the patients were 62.2 ± 14.0 and 62.8 ± 14.5 years in the UVM and SKCM groups, respectively (*P* = 0.790). Of the UVM and SKCM patients, 45 (56.3%) and 42 (62.7%) were male, respectively (*P* = 0.501). There were no significant differences in age and sex between the UVM and SKCM groups.

### Mutational burden and signatures

Mutational burdens identified by whole exome sequencing were significantly higher in SKCM than in UVM patients (*P* < 0.001). The median numbers of mutations were 19 and 321 in the UVM and SKCM patients, respectively. No double nucleotide variant (DNV) was observed in any patients with UVM. The details of mutational burden are presented in Supplementary Table 1.

**Figure 1:**
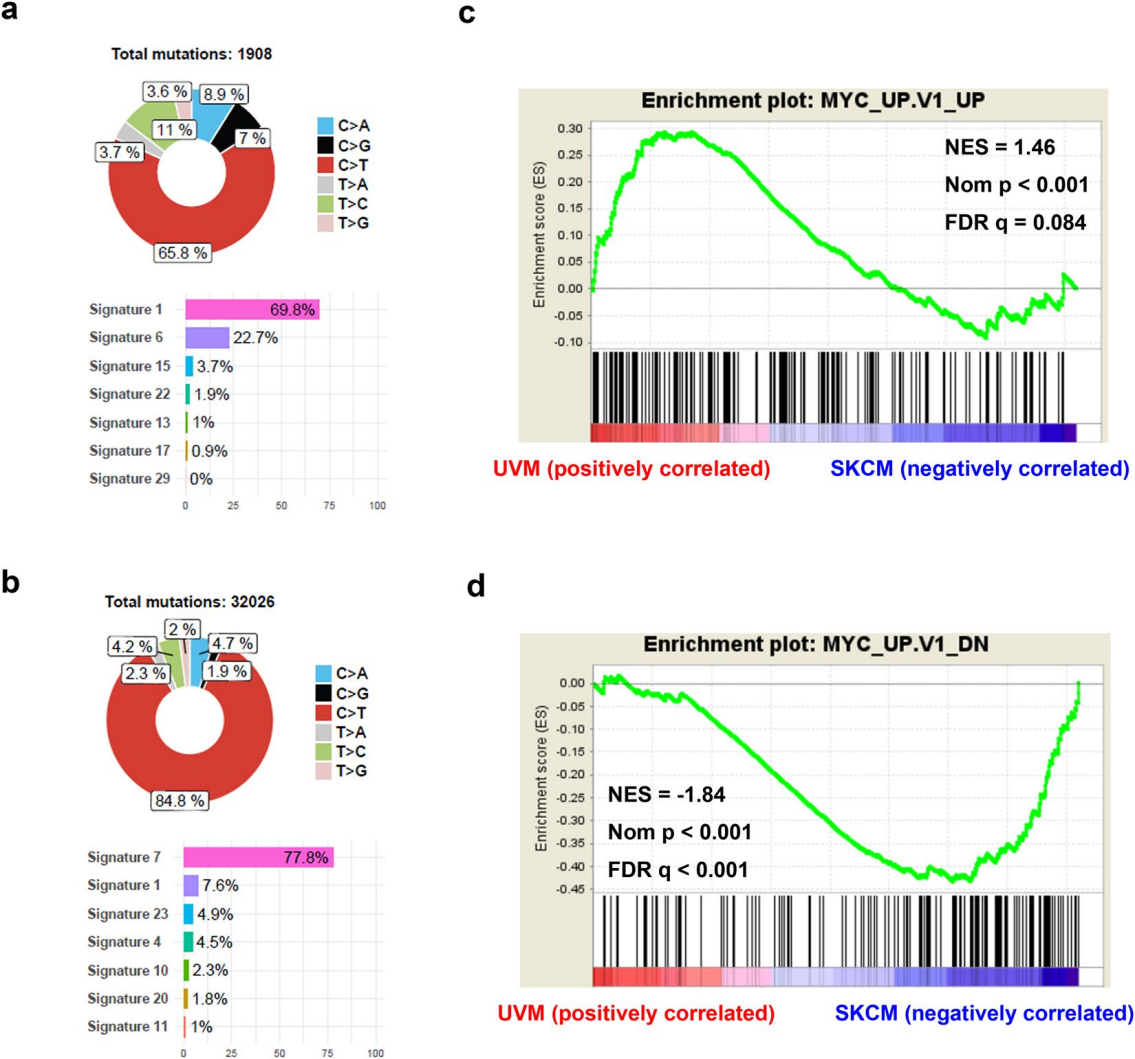
Comparison of exome and transcriptome sequencing data between uveal melanomas and skin cutaneous melanomas. (**a**) Mutational signature analysis for primary uveal melanomas. (**b**) Mutational signature for primary skin cutaneous melanomas. (**c**,**d**) Gene Set Enrichment Analysis plots show upregulation of *MYC* signature genes in primary uveal melanomas when compared with skin cutaneous melanomas. The genes in the genesets are generated from primary epithelial breast cancer cell cultures over-expressing the *MYC* gene.

A total of 1,994 and 32,508 single nucleotide variants (SNVs) and DNVs were identified in 80 UVM and 67 SKCM patients, respectively, and were included in the COSMIC mutational signature analyses. The most common mutational signature identified in UVM was signature 1 (69.8%), which was the result of endogenous mutational processes initiated by spontaneous deamination of 5-methylcytosine (Fig. 1a and Supplementary Fig. 1). Signature 6 (22.7%) and signature 15 (3.7%), which were associated with defective DNA mismatch repair, were also identified in UVM patients. No mutational signature related to ultraviolet light (UV) exposure was found in UVM patients. Regarding SKCM patients, 77.8% were designated as signature 7, which was associated with UV light exposure. Signatures 1, 23, 4, and 10 were also identified in SKCM patients (Fig. 1b and Supplementary Fig. 2). These signatures were likely to be due to tobacco-induced mutations (signature 4) and error-prone polymerase POLE (signature 10). In uveal melanoma, there was no transcriptional strand bias in the somatic mutations (SNVs and DNVs). The somatic mutations were frequently observed in the chromatin regions showing high *DNase I* hypersensitivity (51.6%, indicating regulatory elements) or high GC contents (59.4%).

### MYC upregulation in UVM patients

**Figure 2:**
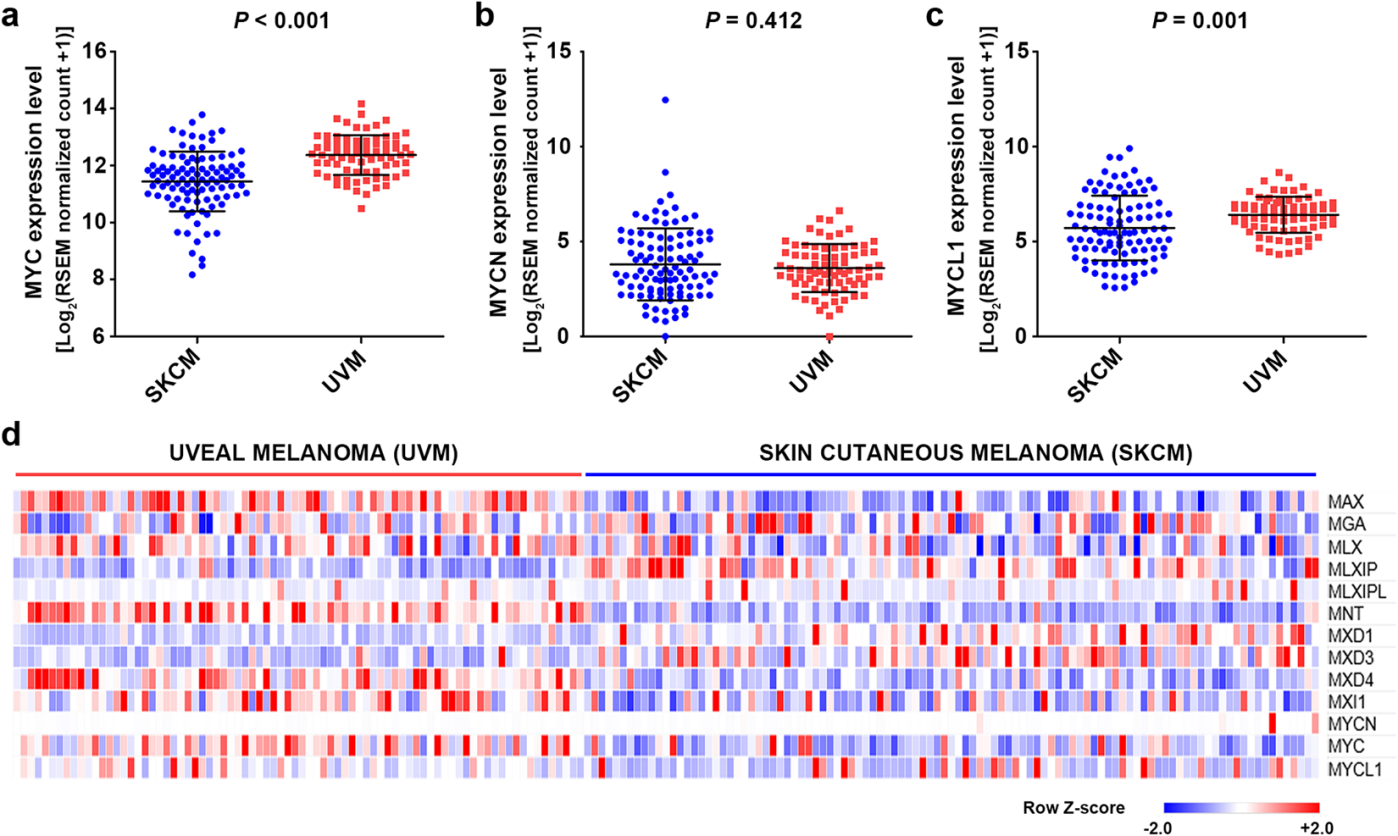
Comparison of the expression levels of *MYC* and its related genes. (**a**–**c**) Comparison of MC, MYCN, and MYCL expressions between uveal melanoma and skin cutaneous melanoma. (**d**) A heat map of the expression levels of *MYC*-related genes in primary uveal melanomas and skin cutaneous melanomas.

Using the transcriptome sequencing data, we performed a gene set enrichment analysis (GSEA) using MSigDB C6 oncogenic signatures to identify the difference in gene expression patterns between UVM and SKCM patients. Most oncogenic signatures including YAP, EGFR, KRAS, BRAF, and MTOR were enriched in SKCM patients (Supplementary Table 2). *MYC* signature genes were significantly enriched in UVM patients (normalized enrichment score = 1.46, normalized p < 0.001, FDR q = 0.084) (Fig. 1c,d). The expression levels of *MYC* (c-myc) and *MYCL1* (l-myc) were higher in UVM patients than in SKCM patients (*P* < 0.001 and *P* = 0.001, respectively). There was no statistically significant difference in the *MYCN* (n-myc) expression level (Fig. 2a–c). When the expression levels of the *MYC* network genes were compared, MAX, MNT, MXD4, MXI1, and MYC expression levels were increased in UVM patients (Fig. 2d).

### MYC copy number variation in UVM patients

**Figure 3:**
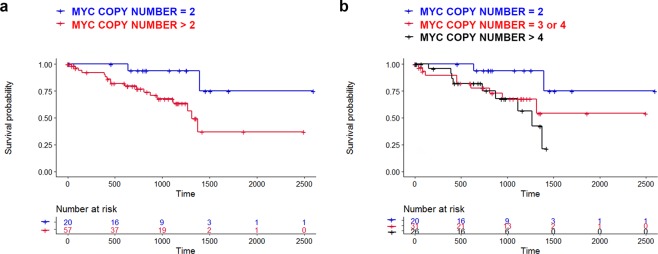
Kaplan-Meier curves showing the association between uveal melanoma-specific survival and *MYC* copy number. (**a**) Comparison between tumors with normal copy number (N = 2) and tumors with high copy number (N > 2). (**b**) Trends showing the survival curves of tumors with various copy numbers.

*MYC* copy number was increased in 59 (73.8%) out of 80 UVM patients. The *MYC* copy number gain was associated with UVM-specific survival (*P* = 0.028; Fig. 3a). A worse UM-specific survival was observed in patients with a larger *MYC* copy number (*P* = 0.028 for trend; Fig. 3b). A high *MYC* copy number was associated with marked pigmentation (*P* < 0.001), high mitotic count (*P* = 0.021), and a large tumor basal diameter (*P* = 0.040). The clinicopathological features of patients with or without gain of the *MYC* copy number are presented in Table 1. Notably, significantly more *BAP1* mutations were observed in patients with high *MYC* copy number (*P* < 0.002). In multivariate cox proportional hazards model, hazard ratios were 2.27 (*P* = 0.201) and 5.45 (*P* = 0.001) for *MYC* copy number gain and *BAP1* mutation, respectively. Harrell’s C indices were 0.623 and 0.687 for for *MYC* copy number gain and *BAP1* mutation, respectively.

**Table 1:** Clinicopathological features of patients with or without gain of *MYC* copy number.

	Normal MYC CN (CN = 2)	High MYC CN (CN > 2)	P-value
Number of Patients	21	59	—
Age, Median (IQR)	60.5 (51.1–72.5)	65.2 (53.3–76.4)	0.240
Male, No. (%)	10 (47.6%)	35 (59.3%)	0.444
AJCC stage			0.511
Stage IIA, No. (%)	2 (9.5%)	2 (3.4%)	
Stage IIB, No. (%)	10 (47.6%)	22 (37.3%)	
Stage IIIA, No. (%)	7 (33.3%)	20 (33.9%)	
Stage IIIB, No. (%)	2 (9.5 %)	8 (13.6%)	
Stage IIIC, No. (%)	0 (0%)	3 (5.1%)	
Stave IV, No. (%)	0 (0%)	4 (6.8%)	
Dominant cell types			
Spindle, No. (%)	18 (85.7%)	39 (66.1%)	0.101
Epithelioid, No. (%)	3 (14.3%)	20 (33.9%)	
Pigmentation			
Minimal, No. (%)	18 (85.7%)	21 (35.6%)	<0.001
Marked, No. (%)	3 (14.3%)	38 (64.4%)	
Mitotic count			
0–5/HPF, No. (%)	21 (100%)	42 (71.2%)	0.021
5–10/HPF, No. (%)	0 (0%)	11 (18.6%)	
>11/HPF, No. (%)	0 (0%)	6 (10.2%)	
Basal diameter(clinical), Median (IQR), mm	15.9 (13.2–17.0)	17.0 (15.1–18.0)	0.040
Basal diameter (pathological), Median (IQR), mm	15.5 (12.0–18.0)	17.0 (15.0–20.0)	0.094
Tumor thickness, Median (IQR), mm	10.0 (8.4–12.0)	11.0 (8.7–12.2)	0.390
GNAQ/11 mutation, No. (%)	19 (90.5%)	55 (93.2%)	0.650
BAP1 mutation, No. (%)	3 (14.3%)	32 (54.2%)	0.002
EIF1AX mutation, No. (%)	10 (47.6%)	0 (0%)	<0.001
SF3B1 mutation, No. (%)	3 (14.3%)	15 (25.4%)	0.373

CN = copy number, IQR = interquartile range, No. = Number AJCC = American Joint Committee on Cancer.

### Kinome-wide siRNA library screening to identify synthetic lethal targets for UVM

**Figure 4:**
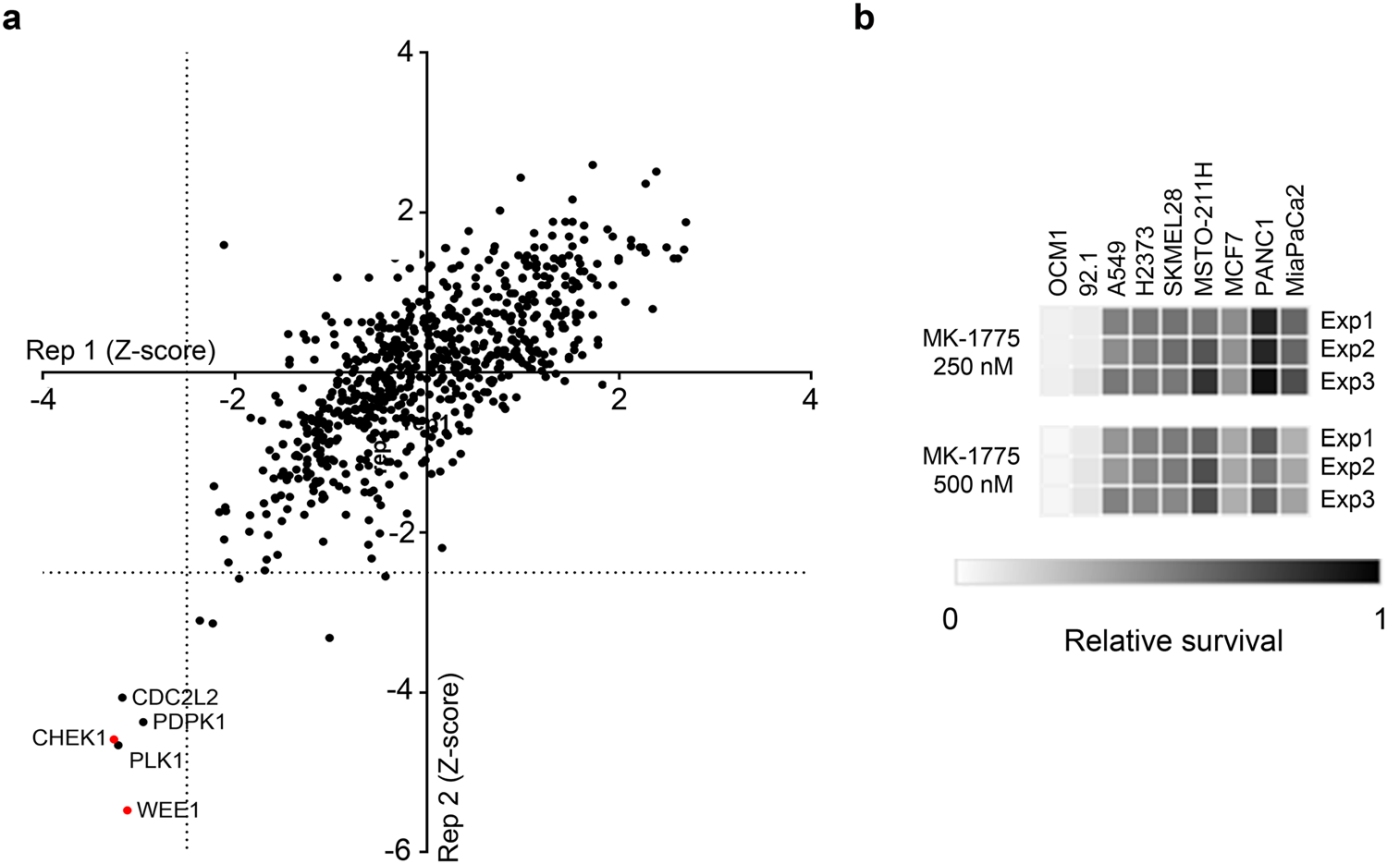
WEE1 as a therapeutic target for uveal melanoma. (**a**) The results of kinome-wide siRNA screening to identify therapeutic targets for uveal melanoma. (**b**) The cell viability of multiple cancer cells treated with the WEE1 inhibitor (MK-1775) at the indicated doses.

Using the 92.1 UVM cell line, we screened a siRNA library of 715 genes across the human kinome to identify synthetic lethal targets for UVM (Fig. 4a and Supplementary Table 3). Pearson’s correlation coefficient between two biological replicates was 0.7425 (*P* < 0.001), indicating little variation. The screen identified five hits (WEE1, PLK1, CHEK1, PDPK1, and CDK11A) that significantly affected survival of 92.1 cells (Z-score < −2.5 in both replicates).

### WEE1 as a therapeutic target for UVM

We added the selective WEE1 inhibitor (MK-1775) at concentrations of 250 or 500 nM to cell lines of multiple human cancers, including UVM (92.1 and OCM1), SKCM (SKMEL28), non-small cell lung cancer (A549), breast cancer (MCF7), mesothelioma (MSTO-211H and H2373), and pancreatic cancer (PANC1 and Mia-PaCa2). Treatment with the WEE1 inhibitor significantly reduced the cell viability of 92.1 and OCM1 UVM cells, compared with other cancer cells (Fig. 4b).

## Discussion

During tumorigenesis, somatic genome mutations occur. SNVs, small insertions/deletions, and copy number variations of the genome alter the function of oncogenes and tumor suppressor genes, and can cause cancer^14^. Intense research efforts regarding cancer genomes have identified several oncogenic pathways important in human cancers, and have led to improved therapeutic outcomes for tumors with EGFR, ALK, and BRAF mutations^15^. Cancer genome sequencing studies using UVM have identified frequent mutations in GNAQ/GNA11^2^. Mutant GNAQ/GNA11-induced activation of the MAPK and Hippo-YAP pathways has been reported in many basic and translational studies, and has suggested as essential for tumorigenesis of UVM^7,16^. However, these studies have not led to an improvement in clinical outcomes of UVM patients. Clinical trials using the MEK1/2 inhibitor, selumetinib, have failed to increase the overall survival of metastatic UVM patients, and drugs targeting the Hippo-YAP pathway are not currently available^5,6^. In addition, metastatic UVM patients have shown intrinsic resistance to various anti-cancer agents including immune checkpoint inhibitors^9^. Thus, an effective treatment of metastatic UVM remains unmet medical needs.

In the present study, we conducted multi-platform omics analyses to identify molecular characteristics and therapeutic targets for UVM. Analysis of whole exome sequencing data revealed that somatic mutations in UVM patients were associated with spontaneous deamination or impaired DNA mismatch repair, but were not associated with UV light. We also identified that most UVMs underwent malignant transformation involving less somatic mutations when compared with SKCMs. Analysis of transcriptome sequencing data suggested a possible reason for this difference. Most oncogenic signatures were high in SKCM patients, whereas the *MYC* signature was significantly higher in UVM patients, which was possibly the result of a *MYC* copy number gain. Even when comparing primary/metastatic SKCM and UVM transcriptomes in the TCGA dataset, the Gene Set Enrichment Analysis (GSEA) of the MYC signature is valid (normalized p-value = 0.006, FDR q-value = 0.146). We believe that the GSEA of the MYC signature comprehensively evaluated changes in many genes affected by MYC, thus better reflecting the activity of MYC in the tumor than the MYC mRNA level or copy number.

*MYC* and its paralogs, *MYCN* and *MYCL*, are well-known oncogenes and frequently amplified in many the human cancers^17^. Following diverse mitogenic and developmental signals, the upregulated and deregulated MYC proteins cause broad changes in gene expressions, resulting in increased cell proliferation^17^. In *MYC*-driven cancers, *MYC* deregulation affects gene expression, DNA replication, or repair processes, leading to oncogenic proliferation^18,19^. In the present study, analyses of whole exome sequencing and transcriptome sequencing data revealed high MYC activity in UVM.

Emerging data have shown that *MYC* activation confers chemoresistance to cancer cells treated with BRAF, MEK, KRAS, or BET inhibitors^20^. Our results showed that the global chemoresistance found in metastatic UVM patients was possibly due to *MYC* deregulation resulting from a gain in copy number. Importantly, *MYC* copy number gain was associated with aggressive pathological features and poor survival outcomes in UVM patients. Because *MYC* deregulation is observed in multiple human cancers and associated with poor prognoses, there have been intense research efforts to develop treatments that target *MYC*^21^. However, direct inhibition of *MYC* is challenging due to the lack of a specific active site, so several methods have been tried to regulate *MYC* indirectly at the level of transcription (BRD4, CDK7, CDK9), translation (mTOR, CPEB), or protein stability (AURKA, PLK1)^21^. Our kinome-wide siRNA library screen also identified BRD4, AURKA, and PLK1 as therapeutic targets in 92.1 UVM cells.

Remarkably, the kinome-wide siRNA library screen identified the G2/M checkpoint proteins, WEE1 and CHEK1, as the strongest therapeutic targets for UVM. They have also been reported to be lethal targets for MYC-driven medulloblastoma, diffuse large B cell lymphoma, breast cancer, and lung cancer^21–23^. A mechanism involving the synthetic lethal effect of WEE1 inhibition on *MYC*-driven cancers includes the possibility that WEE1 enhances survival of cancer cells by protecting the cells from *MYC*-induced replicative stress, and by promoting DNA damage repair^24^. In the present study, a cell viability test using multiple cancer cell lines showed that 92.1 and OCM1 UVM cells were very susceptible to a WEE1 inhibitor (MK-1775) at a clinically tolerable dose.

In multivariate cox proportional hazard model, the hazard ratio was 2.271 for MYC copy number gain. Although the statistical significance of the MYC copy number gain did not come from the multivariate analysis, it cannot be concluded at this time because of small number of tumor-specific death in the TCGA dataset. Considering the Harrell’s C indices for MYC copy number gain and BAP1 mutation, we think that both factors affect survival outcomes of uveal melanoma patients independently. A recent study has shown that 8q gains occur early and subsequently increase during progression in metastatic UVMs using targeted sequencing technology^25^. Interestingly, some cases showed BAP1 mutation in primary tumors, but not in metastases, while high 8q copy number was detected in these metastases. Considering that MYC is located at the 8q24 locus, it is possible that the *MYC* copy number gain is associated with 8q gains in metastatic UVM. These findings may suggest that 8q gain and *MYC* copy number gain have distinct roles in metastasis apart from BAP1 mutations.

This study used exome and transcriptome data to elucidate the mechanism by which metastatic UVMs acquired chemoresistance. Combined with a kinome-wide siRNA library screen, our results identified UVM as a *MYC*-driven cancer, with several therapeutic targets. The G2/M checkpoint inhibitors are currently in clinical trials for treatment of various *MYC*-driven cancers, including lung cancer (NCT02688907), lymphoma, and ovarian cancer^23,26^. Based on the present study, we expect that WEE1 inhibition will ameliorate intrinsic chemoresistance and increase the survival of metastatic UVM patients.

## Methods

### Study population and acquisition of TCGA data

The study population included primary UVM and SKCM patients in the TCGA dataset. Whole exome sequencing data and transcriptome sequencing data were obtained from Firebrowse (Broad Institute). The most recent follow-up survival data and clinicopathological information, including age, sex, tumor stage, pigmentation, mitotic count, tumor basal diameter, and thickness were downloaded using the R package TCGAbiolinks (last update: 2018-09-06; http://bioconductor.org/packages/release/bioc/html/TCGAbiolinks.html). All samples in TCGA have been collected and utilized following strict human subject protection guidelines, informed consent, and institutional review board review of the protocols.

### Mutation identification and mutational signature analysis

Whole exome sequencing data (Mutation Packager Oncotated Calls; downloaded from https://gdac.broadinstitute.org) were used for identification of SNVs, DNVs, insertions, and deletions. SNV and DNV indicate a somatic variation in a single nucleotide or consecutive two nucleotides in tumor cells. For the analysis of somatic DNA mutations with DNA sequence contexts, we used a public web service program called MUTALISK (http://mutalisk.org)^27^. Mutational processes in tumors were estimated according to the COSMIC mutational signature^28^. The characteristics of the genome regions corresponding to somatic mutations (SNV and DNV) were also analyzed using MUTALISK. For DNase I hypersensitivity analysis, the 1-megabase interval chromosome region was categorized into a low (≤25th percentile), intermediate (25th–75th percentiles), or high (≥75th percentile) level based on the normalized read intensity. For GC content analysis, the ratios of the guanine (G) and cytosine (C) nucleotides were calculated in each 1 kilobase window. Subsequently, each bin was labeled as a low (≤25th percentile), intermediate (25th–75th percentile), or high (≥75th percentile) level. The detailed methods for each analysis were published elsewhere^27^.

### Transcriptome analysis and MYC copy number variations

The mRNA expression (RNA Seq V2, RSEM) data were obtained from TCGA datasets for primary UVM and SKCM patients. Gene Set Enrichment Analysis (GSEA) was performed using the C6 MSigDB gene set database to identify gene sets enriched in primary UVMs^29^. The gene sets for the MYC signature used in this study included the 200 genes that were significantly upregulated (MYC_UP.V1_UP) or downregulated (MYC_UP.V1_DN) in primary epithelial breast cancer cell cultures overexpressing the *c-MYC* gene. Heatmaps were generated using Morpheus (Broad institute; https://software.broadinstitute.org/morpheus/). The *MYC* copy number of each UVM patient was derived from previous studies using the FACET algorithms for calculations^2^.

### Kinome-wide siRNA library screening

A kinome-wide siRNA library targeting 715 human kinases (Dharmacon, Lafayette, CO, USA) was used. Four different siRNAs targeting each kinase gene were spotted as a pool on 384-well plates (CELLSTAR; Greiner Bio-One, Monroe, NC, USA) using a Biomek FX Laboratory Automation Workstation (Beckman Coulter, Brea, CA, USA). Lipofectamine RNAiMAX (Invitrogen, Carlsbad, CA, USA) dissolved in Opti-MEM (Gibco, Gaithersburg, MD, USA) was added to the assay plates to perform reverse transfection. The final total siRNA concentration was 15 nM (3.75 nM for each siRNA targeting each kinase gene). The 92.1 cells were suspended and seeded onto the assay plates using a MultiFlo Microplate Dispenser (BioTek, Winnoski, VT, USA) at 850 cells/well with a final culture volume of 50 μL. The cells were incubated for 120 h, and then Cell Counting Kit-8 reagent (CCK8; Dojindo Molecular Technologies, Rockville, MD, USA) was added for 2 h to measure the cell viability. Viable cells were quantitated by measurement of the absorbance at 450 nm. Genes with Z-scores < −2.5 were considered therapeutic targets.

### Cell viability assay

MK-1775 (Adavosertib; Selleckchem, Houston TX, USA) was used to evaluate the effect of the WEE1 inhibition on the viability of cancer cells. Multiple cancer cells were incubated for 24 h after plating, and then treated with WEE1 inhibitor for 72 h. Viable cells were quantitated using the CCK8 reagent.

### Statistical analysis

The clinicopathological characteristics were compared using the chi-square or Fisher’s exact test for categorical variables. Student’s *t-*tests and the Mann-Whitney U test were used to compare two groups for continuous variables with and without normal distribution, respectively. The Kaplan-Meier method and the log-rank test were used for tumor-specific survival analyses. Cox proportional hazard model and Harrell’s C index were also used for survival analysis. A value of P < 0.05 was considered to indicate statistical significance.

## Supplementary information

Supplementary Materials
