# Supplementary material for: Multi-Platform Omics Analysis for Identification of Molecular Characteristics and Therapeutic Targets of Uveal Melanoma
Source: Sci Rep. 2019 Dec 17;9:19235. doi: 10.1038/s41598-019-55513-z (PMC6917695; doi:10.1038/s41598-019-55513-z)
Supplement: Supplementary file 1 — Supplementary Materials [file 41598_2019_55513_MOESM1_ESM.pdf]

Supplementary Materials for:

Multi-Platform Omics Analysis for Identification of Molecular Characteristics and Therapeutic Targets of Uveal Melanoma

Yong Joon Kim[1] Seo Jin Park[1], Kyung Joo Maeng[1], Sung Chul Lee[1], Christopher Seungkyu Lee[1,2]

[1]Department of Ophthalmology, Institute of Vision Research, Severance Hospital, Yonsei University College of Medicine, Seoul, Republic of Korea.

[2]Department of Ophthalmology, Institute of Vision Research, Gangnam Severance Hospital, Yonsei University College of Medicine, Seoul, Republic of Korea.

Supplementary Materials

Supplementary Figure 1. Detailed results of the mutational signature decomposition of uveal melanoma samples.

Supplementary Figure 2. Detailed results of the mutational signature decomposition of skin cutaneous melanoma samples.

Supplementary Table 1. The number of mutations identified by whole exome sequencing in uveal melanomas and skin cutaneous melanomas

Supplementary Table 2. The results of Gene Set Enrichment Analysis using transcriptomes of primary uveal melanomas and skin cutaneous melanomas.

Supplementary Table 3. The results of kinome-wide siRNA screen (two biological replicates).

Supplementary Figure 1. Detailed results of the mutational signature decomposition of uveal melanoma samples.

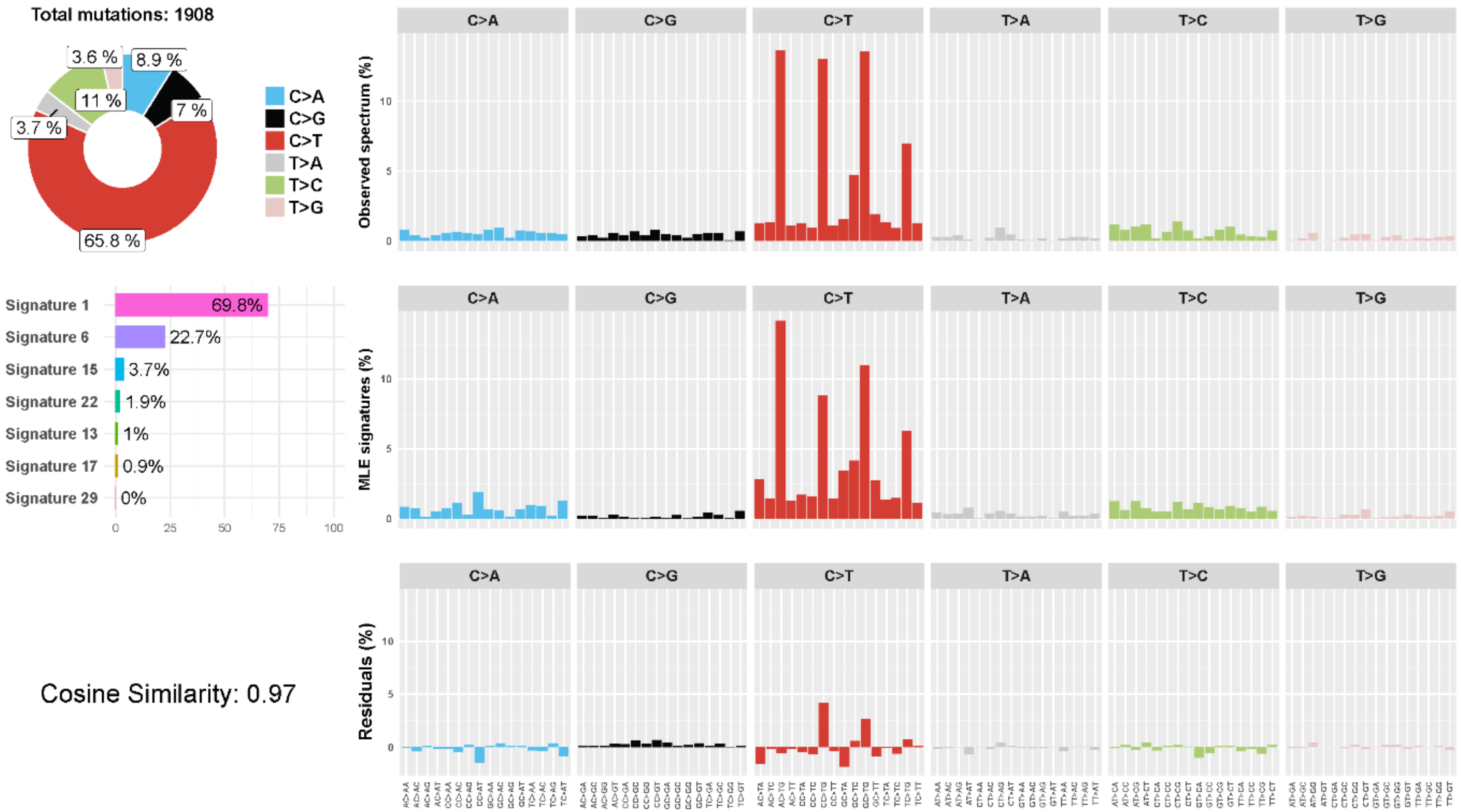

Supplementary Figure 2. Detailed results of the mutational signature decomposition of skin cutaneous melanoma samples.

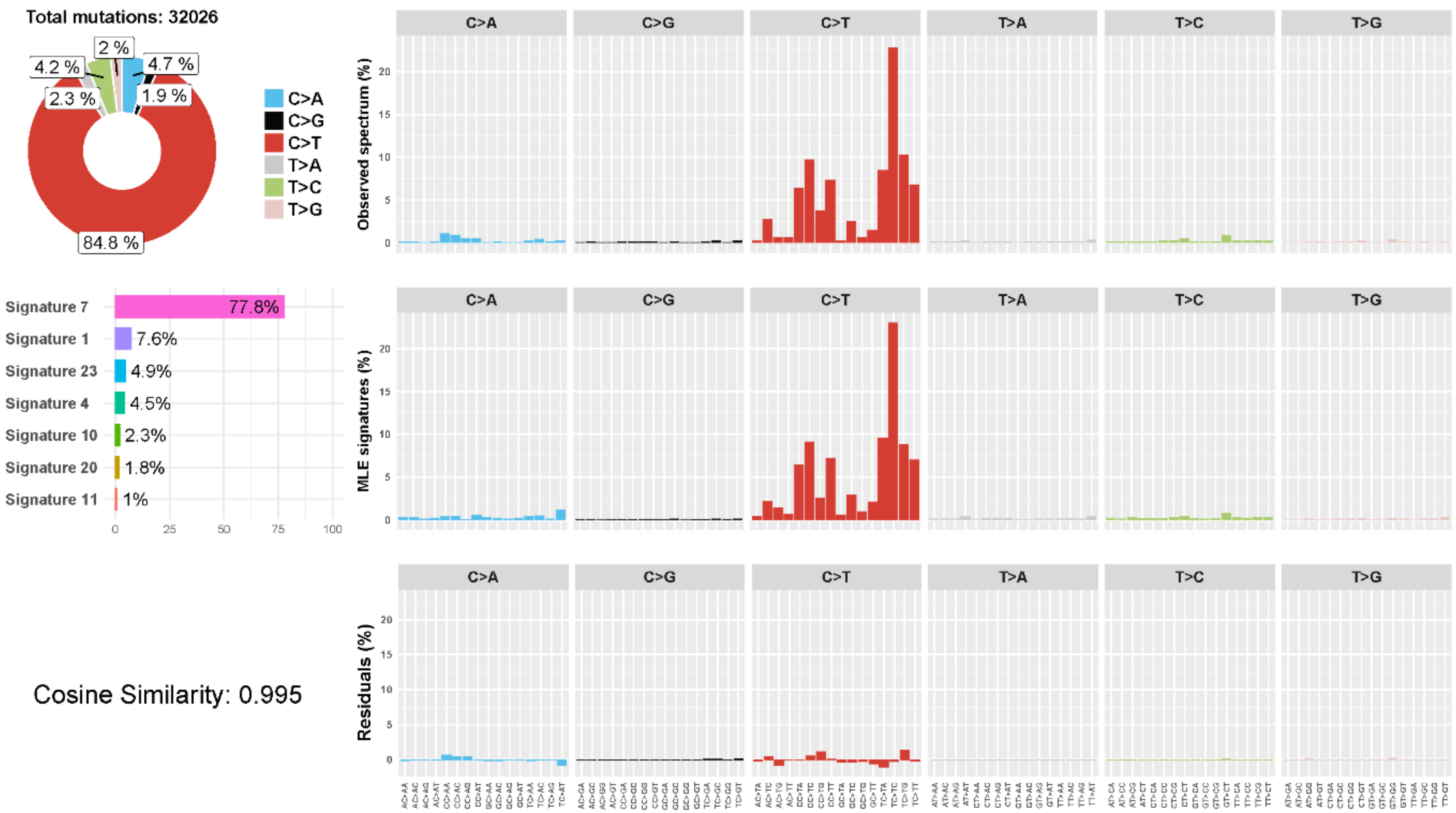

Supplementary Table 1. The number of mutations identified by whole exome sequencing in uveal melanomas and skin cutaneous melanomas

	UVM	SKCM	P-value
Number of patients	80	67	-
Total mutations	19.0 (16.0–25.0)	321.0 (91.0-620.0)	< 0.001
SNV	17.0 (14.0–22.8)	285.0 (84.0–588.0)	< 0.001
DNV	0.0 (0.0–0.0)	11.0 (2.0–20.0)	< 0.001
INS	0.0 (0.0–1.0)	1.0 (0.0–2.0)	< 0.001
DEL	2.0 (1.0–3.0)	4.0 (2.0–6.0)	< 0.001

UVM = uveal melanoma, SKCM = Skin cutaneous melanoma, SNV = Single nucleotide variants, DNV = double nucleotide variants, INS = insertion, DEL = deletion
Data are presented in median (interquartile range)

Supplementary Table 2. The results of Gene Set Enrichment Analysis using transcriptomes of primary uveal melanomas and skin cutaneous melanomas.

NAME	NES	NOM p-value	FDR q-value	RANK at MAX
Gene Sets Enriched in Uveal melanoma				
VEGF_A_UP.V1_UP	1.471	<0.001	0.155	3096
MYC_UP.V1_UP	1.464	<0.001	0.084	4237
SIRNA_EIF4GI_UP	1.393	0.027	0.114	2237
RAPA_EARLY_UP.V1_DN	1.373	<0.001	0.106	2613
MTOR_UP.V1_DN	1.336	0.011	0.121	2036
Gene Sets Enriched in Skin Cutaneous Melanoma				
VEGF_A_UP.V1_DN	-2.480	<0.001	<0.001	3905
SIRNA_EIF4GI_DN	-2.384	<0.001	<0.001	4390
RB_P107_DN.V1_UP	-2.383	<0.001	<0.001	2875
CSR_LATE_UP.V1_UP	-2.352	<0.001	<0.001	3073
CORDENONSI_YAP_CONSERVED_SIGNATURE	-2.198	<0.001	<0.001	4529
EGFR_UP.V1_UP	-2.166	<0.001	<0.001	5430
ERB2_UP.V1_DN	-2.129	<0.001	<0.001	6070
RPS14_DN.V1_DN	-2.106	<0.001	<0.001	3041
TBK1.DF_DN	-2.096	<0.001	<0.001	4746
GCNP_SHH_UP_LATE.V1_UP	-2.074	<0.001	<0.001	4681
RAF_UP.V1_UP	-2.072	<0.001	<0.001	4926
CSR_EARLY_UP.V1_UP	-2.032	<0.001	<0.001	4661
TBK1.DF_UP	-1.992	<0.001	<0.001	4459

MEL18_DN.V1_UP	-1.978	<0.001	<0.001	7362
BMI1_DN_MEL18_DN.V1_UP	-1.953	<0.001	<0.001	7414
GCNP_SHH_UP_EARLY.V1_UP	-1.952	<0.001	<0.001	3173
SINGH_KRAS_DEPENDENCY_SIGNATURE_	-1.951	<0.001	<0.001	5842
HOXA9_DN.V1_DN	-1.946	<0.001	<0.001	3349
RPS14_DN.V1_UP	-1.914	<0.001	<0.001	6499
PRC2_EZH2_UP.V1_DN	-1.912	<0.001	<0.001	3987
STK33_SKM_UP	-1.901	<0.001	<0.001	5485
PRC2_EZH2_UP.V1_UP	-1.884	<0.001	<0.001	4765
KRAS.DF.V1_UP	-1.872	<0.001	<0.001	4710
RB_P130_DN.V1_DN	-1.861	<0.001	<0.001	6289
IL15_UP.V1_UP	-1.860	<0.001	<0.001	5533
E2F3_UP.V1_UP	-1.848	<0.001	<0.001	3316
MYC_UP.V1_DN	-1.836	<0.001	<0.001	5301
TGFB_UP.V1_UP	-1.825	<0.001	<0.001	5509
IL2_UP.V1_UP	-1.822	<0.001	<0.001	5440
PIGF_UP.V1_UP	-1.822	<0.001	<0.001	6998
BMI1_DN.V1_UP	-1.822	<0.001	<0.001	7236
SRC_UP.V1_DN	-1.820	<0.001	<0.001	5066
STK33_UP	-1.793	<0.001	0.001	5410
HINATA_NFKB_IMMU_INF	-1.786	0.003	0.001	3699
HOXA9_DN.V1_UP	-1.783	<0.001	0.001	6220
STK33_NOMO_UP	-1.782	<0.001	0.001	4879
LTE2_UP.V1_DN	-1.778	<0.001	0.001	6941
E2F1_UP.V1_UP	-1.766	<0.001	0.001	3670
SNF5_DN.V1_UP	-1.758	<0.001	0.001	4747

BCAT_GDS748_UP	-1.729	0.003	0.001	4656
CAHOY_OLIGODENDROCUTIC	-1.717	<0.001	0.001	5389
MTOR_UP.V1_UP	-1.708	0.001	0.001	3865
TBK1.DN.48HRS_DN	-1.702	0.003	0.001	4900
KRAS.LUNG.BREAST_UP.V1_UP	-1.701	<0.001	0.001	6364
ESC_V6.5_UP_EARLY.V1_DN	-1.651	<0.001	0.003	6784
MEK_UP.V1_DN	-1.640	0.000	0.003	6941
AKT_UP.V1_UP	-1.637	<0.001	0.003	5284
P53_DN.V1_UP	-1.632	0.000	0.003	6669
MTOR_UP.N4.V1_UP	-1.632	<0.001	0.003	4775
TBK1.DN.48HRS_UP	-1.627	0.007	0.003	1845
EGFR_UP.V1_DN	-1.625	0.001	0.003	4844
RELA_DN.V1_DN	-1.619	<0.001	0.003	6932
AKT_UP_MTOR_DN.V1_UP	-1.594	<0.001	0.005	5284
MEK_UP.V1_UP	-1.570	<0.001	0.006	4229
E2F1_UP.V1_DN	-1.560	<0.001	0.007	4705
CTIP_DN.V1_DN	-1.553	0.001	0.008	6971
KRAS.BREAST_UP.V1_UP	-1.545	0.004	0.009	6596
ATF2_S_UP.V1_UP	-1.541	<0.001	0.009	3588
PRC2_EED_UP.V1_DN	-1.483	0.011	0.019	2997
KRAS.600.LUNG.BREAST_UP.V1_UP	-1.482	0.000	0.019	6377
BCAT_BILD_ET_AL_DN	-1.481	0.042	0.019	4681
ESC_V6.5_UP_LATE.V1_DN	-1.472	0.002	0.021	3259
RB_P107_DN.V1_DN	-1.466	0.015	0.022	4619
SNF5_DN.V1_DN	-1.459	0.005	0.023	4656
CRX_DN.V1_DN	-1.452	0.017	0.025	4272

RAF_UP.V1_DN	-1.447	0.011	0.026	4614
PDGF_UP.V1_UP	-1.426	0.011	0.033	3096
KRAS.LUNG_UP.V1_UP	-1.423	0.015	0.034	6104
ESC_J1_UP_LATE.V1_UP	-1.414	0.008	0.037	4881
NRL_DN.V1_DN	-1.414	0.014	0.036	5749
CAHOY_ASTROGLIAL	-1.410	0.024	0.037	4890
LEF1_UP.V1_UP	-1.401	0.012	0.040	6684
KRAS.300_UP.V1_UP	-1.392	0.021	0.044	5148
LTE2_UP.V1_UP	-1.390	0.016	0.045	3863
ATF2_UP.V1_UP	-1.382	0.020	0.048	3026
RB_DN.V1_UP	-1.379	0.025	0.049	3541
CRX_NRL_DN.V1_DN	-1.343	0.044	0.071	5087
JNK_DN.V1_UP	-1.329	0.028	0.082	7498
CAMP_UP.V1_DN	-1.328	0.036	0.081	4478
JNK_DN.V1_DN	-1.326	0.027	0.082	5067
RB_DN.V1_DN	-1.322	0.037	0.084	3206
ATM_DN.V1_DN	-1.321	0.035	0.084	6199
ESC_J1_UP_EARLY.V1_DN	-1.318	0.039	0.085	3541
PRC1_BMI_UP.V1_UP	-1.310	0.027	0.092	7577
KRAS.LUNG_UP.V1_DN	-1.309	0.043	0.092	8033
ATF2_UP.V1_DN	-1.297	0.046	0.100	7677
PRC2_SUZ12_UP.V1_DN	-1.297	0.048	0.099	7052
KRAS.600_UP.V1_UP	-1.283	0.035	0.112	5301
NFE2L2.V2	-1.260	0.020	0.131	6409

Supplementary Table 3. The results of kinome-wide siRNA screen (two biological replicates).

Number	Rank	Gene ID	Gene Symbol	Relative cell viability (Z-score)		
				**Replicate 1**	**Replicate 2**	**Average**
461	1	7465	WEE1	-3.120	-5.477	-4.299
25	2	5347	PLK1	-3.216	-4.660	-3.938
264	3	1111	CHEK1	-3.261	-4.589	-3.925
698	4	5170	PDPK1	-2.955	-4.371	-3.663
266	5	728642	CDC2L2	-3.172	-4.064	-3.618
256	6	5296	PIK3R2	-2.366	-3.102	-2.734
322	7	9212	AURKB	-2.232	-3.138	-2.685
608	8	27010	TPK1	-1.958	-2.576	-2.267
374	9	51755	CRK7	-2.069	-2.372	-2.220
566	10	200576	PIP5K3	-1.015	-3.319	-2.167
278	11	6790	AURKA	-2.112	-2.085	-2.099
707	12	2932	GSK3B	-1.689	-2.472	-2.080
563	13	5207	PFKFB1	-1.671	-2.339	-2.005
515	14	81788	SNARK	-2.164	-1.744	-1.954
269	15	984	CDC2L1	-1.851	-1.987	-1.919
406	16	701	BUB1B	-1.558	-2.279	-1.919
454	17	23476	BRD4	-2.094	-1.731	-1.912
286	18	5590	PRKCZ	-2.101	-1.683	-1.892
353	19	6731	SRP72	-1.653	-2.029	-1.841
513	20	6872	TAF1	-2.219	-1.420	-1.819
400	21	65220	FLJ13052	-1.844	-1.781	-1.813
275	22	4593	MUSK	-1.674	-1.770	-1.722
324	23	2868	GRK4	-1.512	-1.856	-1.684
265	24	8631	SCAP1	-1.699	-1.632	-1.666
564	25	3645	INSRR	-1.081	-2.112	-1.597
404	26	5600	MAPK11	-1.456	-1.707	-1.581
354	27	9162	DGKI	-1.578	-1.582	-1.580
246	28	2045	EPHA7	-1.583	-1.564	-1.573
434	29	55359	STYK1	-1.522	-1.534	-1.528
648	30	29959	NRBP	-1.277	-1.777	-1.527
600	31	11344	PTK9L	-0.434	-2.548	-1.491
447	32	156	ADRBK1	-1.428	-1.552	-1.490
283	33	8503	PIK3R3	-1.708	-1.221	-1.464
285	34	65267	PRKWNK3	-1.596	-1.323	-1.460

565	35	1740	DLG2	-0.577	-2.324	-1.451
482	36	29922	NME7	-1.207	-1.636	-1.421
616	37	9475	ROCK2	-1.520	-1.287	-1.403
253	38	3932	LCK	-1.460	-1.343	-1.402
481	39	26750	RPS6KC1	-0.610	-2.151	-1.381
243	40	51701	NLK	-1.499	-1.233	-1.366
445	41	5597	MAPK6	-1.433	-1.284	-1.358
610	42	415116	PIM3	-1.089	-1.582	-1.335
46	43	8844	KSR	-1.340	-1.326	-1.333
487	44	4752	NEK3	-1.181	-1.481	-1.331
644	45	23049	SMG1	-1.070	-1.560	-1.315
126	46	4882	NPR2	-1.576	-1.048	-1.312
508	47	5589	PRKCSH	-0.846	-1.777	-1.311
308	48	23636	NUP62	-1.086	-1.522	-1.304
364	49	5631	PRPS1	-1.640	-0.968	-1.304
485	50	2263	FGFR2	-1.185	-1.420	-1.302
433	51	2081	ERN1	-1.360	-1.212	-1.286
323	52	3985	LIMK2	-1.249	-1.305	-1.277
624	53	3643	INSR	-1.159	-1.355	-1.257
483	54	4294	MAP3K10	-1.137	-1.377	-1.257
244	55	5209	PFKFB3	-1.235	-1.269	-1.252
567	56	657	BMPR1A	-0.492	-2.011	-1.252
372	57	2268	FGR	-1.215	-1.266	-1.240
329	58	4598	MVK	-1.256	-1.224	-1.240
592	59	57144	PAK7	-1.111	-1.355	-1.233
488	60	10087	COL4A3BP	-0.607	-1.845	-1.226
367	61	7297	TYK2	-1.372	-1.039	-1.206
606	62	5166	PDK4	-1.836	-0.569	-1.203
689	63	673	BRAF	-1.501	-0.897	-1.199
279	64	10298	PAK4	-1.249	-1.144	-1.196
426	65	5127	PCTK1	-1.358	-1.027	-1.193
443	66	2580	GAK	-1.335	-1.048	-1.192
355	67	1445	CSK	-0.974	-1.367	-1.171
493	68	1946	EFNA5	-0.916	-1.420	-1.168
344	69	197258	FUK	-1.247	-1.084	-1.165
365	70	1453	CSNK1D	-1.721	-0.604	-1.163
327	71	10020	GNE	-0.840	-1.466	-1.153
298	72	1606	DGKA	-1.279	-0.950	-1.114
277	73	79012	MGC8407	-1.095	-1.132	-1.113
664	74	8899	PRPF4B	-1.347	-0.869	-1.108

427	75	4216	MAP3K4	-1.038	-1.174	-1.106
436	76	8395	PIP5K1B	-1.111	-1.090	-1.100
486	77	2065	ERBB3	-0.179	-2.011	-1.095
437	78	1716	DGUOK	-1.254	-0.935	-1.094
306	79	283455	KSR2	-1.142	-1.019	-1.080
84	80	3654	IRAK1	-0.652	-1.495	-1.074
366	81	8444	DYRK3	-1.499	-0.646	-1.072
122	82	2475	FRAP1	-0.484	-1.659	-1.072
435	83	9891	ARK5	-1.113	-1.022	-1.067
267	84	6788	STK3	-1.038	-1.084	-1.061
501	85	4915	NTRK2	-0.846	-1.265	-1.055
363	86	28996	HIPK2	-1.449	-0.655	-1.052
208	87	1436	CSF1R	-0.593	-1.498	-1.046
195	88	2064	ERBB2	-0.513	-1.573	-1.043
276	89	9706	ULK2	-1.111	-0.968	-1.039
87	90	4217	MAP3K5	-0.992	-1.084	-1.038
405	91	5232	PGK2	-0.970	-1.102	-1.036
4	92	1760	DMPK	-1.156	-0.908	-1.032
561	93	5583	PRKCH	0.156	-2.191	-1.018
196	94	5571	PRKAG1	-0.559	-1.475	-1.017
287	95	3702	ITK	-1.247	-0.768	-1.007
361	96	57761	TRIB3	-0.872	-1.141	-1.006
10	97	816	CAMK2B	-0.647	-1.364	-1.006
247	98	140901	STK35	-1.140	-0.846	-0.993
705	99	4638	MYLK	-0.209	-1.762	-0.986
425	100	388228	SBK1	-1.254	-0.703	-0.978
294	101	157285	DKFZP761P0423	-1.249	-0.703	-0.976
473	102	1841	DTYMK	-1.081	-0.858	-0.969
348	103	369	ARAF1	-1.172	-0.750	-0.961
526	104	1460	CSNK2B	-1.542	-0.379	-0.960
274	105	65018	PINK1	-1.024	-0.893	-0.959
444	106	6196	RPS6KA2	-1.283	-0.631	-0.957
668	107	5230	PGK1	-1.133	-0.775	-0.954
345	108	205	AK3	-1.161	-0.735	-0.948
684	109	169436	C9ORF96	-1.081	-0.807	-0.944
245	110	10420	TESK2	-0.704	-1.179	-0.942
667	111	5394	EXOSC10	-1.578	-0.292	-0.935
466	112	4215	MAP3K3	-1.006	-0.864	-0.935
293	113	91461	LOC91461	-1.120	-0.744	-0.932
514	114	23097	CDK11	-1.310	-0.533	-0.922

356	115	8518	IKBKAP	-1.269	-0.566	-0.918
431	116	64080	RBKS	-0.949	-0.858	-0.903
346	117	1459	CSNK2A2	-1.195	-0.610	-0.902
568	118	221823	PRPS1L1	-0.393	-1.398	-0.896
376	119	29993	PACSIN1	-1.136	-0.646	-0.891
358	120	22858	ICK	-1.206	-0.574	-0.890
357	121	157	ADRBK2	-0.695	-1.081	-0.888
97	122	5581	PRKCE	-0.609	-1.162	-0.885
273	123	5576	PRKAR2A	-0.933	-0.828	-0.881
714	124	6300	MAPK12	-0.500	-1.240	-0.870
268	125	2324	FLT4	-1.117	-0.619	-0.868
507	126	5894	RAF1	-1.026	-0.710	-0.868
544	127	1025	CDK9	-0.640	-1.092	-0.866
296	128	84197	FLJ23356	-1.063	-0.661	-0.862
326	129	5613	PRKX	-1.076	-0.646	-0.861
125	130	9262	STK17B	-1.133	-0.583	-0.858
325	131	340156	LOC340156	-1.086	-0.610	-0.848
654	132	115701	HAK	-1.023	-0.642	-0.832
375	133	1027	CDKN1B	-1.122	-0.527	-0.824
45	134	64327	C7ORF2	-0.613	-1.007	-0.810
328	135	5575	PRKAR1B	-0.927	-0.667	-0.797
575	136	117283	IHPK3	-1.081	-0.458	-0.770
81	137	5290	PIK3CA	-0.416	-1.114	-0.765
652	138	2242	FES	-0.242	-1.287	-0.764
83	139	55577	NAGK	-0.384	-1.114	-0.749
677	140	1120	CHKB	-1.118	-0.379	-0.748
206	141	4354	MPP1	-1.297	-0.199	-0.748
403	142	203054	ADCK5	-1.024	-0.455	-0.740
423	143	8573	CASK	-0.965	-0.512	-0.739
424	144	9149	DYRK1B	-1.024	-0.446	-0.735
682	145	1030	CDKN2B	-1.089	-0.379	-0.734
377	146	1613	DAPK3	-0.827	-0.610	-0.718
35	147	64122	FN3K	-0.302	-1.135	-0.718
289	148	27148	STK36	-0.886	-0.542	-0.714
304	149	284086	NEK8	-0.588	-0.822	-0.705
8	150	5747	PTK2	-0.570	-0.837	-0.703
422	151	4058	LTK	-0.927	-0.479	-0.703
428	152	23012	STK38L	-0.813	-0.574	-0.694
446	153	131890	GRK7	-0.809	-0.574	-0.692
359	154	90956	ADCK2	-0.761	-0.604	-0.683

371	155	5159	PDGFRB	-0.704	-0.661	-0.682
441	156	7048	TGFBR2	-0.654	-0.703	-0.678
297	157	5165	PDK3	-0.840	-0.512	-0.676
387	158	5566	PRKACA	-0.695	-0.640	-0.668
524	159	56848	SPHK2	-1.435	0.101	-0.667
295	160	25778	DUSTYPK	-1.006	-0.306	-0.656
416	161	9641	IKBKE	-0.820	-0.488	-0.654
626	162	23235	SIK2	-0.695	-0.606	-0.650
88	163	5562	PRKAA1	-0.541	-0.756	-0.648
674	164	8527	DGKD	-1.431	0.158	-0.636
281	165	375449	MAST4	-0.616	-0.655	-0.635
3	166	2046	EPHA8	-0.432	-0.825	-0.628
457	167	5634	PRPS2	-0.895	-0.360	-0.627
194	168	11284	PNKP	-0.636	-0.598	-0.617
288	169	84254	CAMKK1	-0.986	-0.247	-0.616
1	170	7272	TTK	-0.452	-0.780	-0.616
347	171	7444	VRK2	-0.743	-0.479	-0.611
562	172	5063	PAK3	0.045	-1.265	-0.610
242	173	10000	AKT3	-0.747	-0.455	-0.601
34	174	3099	HK2	-0.214	-0.986	-0.600
438	175	79646	PANK3	-0.620	-0.566	-0.593
114	176	79858	NEK11	-0.050	-1.129	-0.589
442	177	23617	STK22B	-0.827	-0.351	-0.589
384	178	83694	RPS6KL1	-0.881	-0.285	-0.583
193	179	2260	FGFR1	-0.802	-0.357	-0.579
86	180	255239	ANKK1	-0.377	-0.774	-0.576
530	181	57147	PACE-1	-0.367	-0.775	-0.571
368	182	53904	MYO3A	-0.886	-0.247	-0.566
248	183	6794	STK11	-0.570	-0.560	-0.565
341	184	3705	ITPK1	-0.679	-0.446	-0.563
462	185	1021	CDK6	-0.654	-0.464	-0.559
496	186	160851	DGKH	-0.544	-0.569	-0.557
480	187	5608	MAP2K6	-0.018	-1.092	-0.555
448	188	8558	CDK10	-0.597	-0.512	-0.555
671	189	1949	EFNB3	-0.334	-0.775	-0.554
280	190	5594	MAPK1	-0.761	-0.342	-0.551
503	191	5582	PRKCG	-0.172	-0.926	-0.549
36	192	51232	CRIM1	0.138	-1.236	-0.549
451	193	2042	EPHA3	-0.650	-0.446	-0.548
517	194	5565	PRKAB2	-0.934	-0.155	-0.545

484	195	83549	UCK1	-0.463	-0.606	-0.534
307	196	5573	PRKAR1A	-0.747	-0.315	-0.531
362	197	80122	FLJ23074	-0.597	-0.455	-0.526
388	198	2185	PTK2B	-0.654	-0.387	-0.520
262	199	4140	MARK3	-0.754	-0.285	-0.520
455	200	7006	TEC	-0.936	-0.104	-0.520
596	201	139728	PNCK	-0.919	-0.108	-0.514
584	202	9064	MAP3K6	-0.735	-0.292	-0.514
586	203	440275	EIF2AK4	-1.174	0.158	-0.508
282	204	1160	CKMT2	-0.579	-0.428	-0.504
453	205	9807	IHPK1	-0.570	-0.437	-0.504
456	206	2395	FRDA	-0.734	-0.268	-0.501
665	207	8780	RIOK3	-0.783	-0.202	-0.493
605	208	51776	ZAK	-1.446	0.479	-0.483
645	209	51447	IHPK2	-0.665	-0.292	-0.479
686	210	2585	GALK2	-0.838	-0.108	-0.473
430	211	5569	PKIA	-0.504	-0.437	-0.471
704	212	54822	TRPM7	0.016	-0.955	-0.470
257	213	3656	IRAK2	-0.454	-0.470	-0.462
458	214	26289	AK5	-0.629	-0.294	-0.462
527	215	2066	ERBB4	-0.916	-0.007	-0.462
385	216	27330	RPS6KA6	-0.584	-0.324	-0.454
378	217	5295	PIK3R1	-0.566	-0.333	-0.449
44	218	7371	UMPK	-0.320	-0.574	-0.447
2	219	6850	SYK	-0.493	-0.384	-0.438
241	220	56164	STK31	-0.516	-0.360	-0.438
472	221	22853	LMTK2	-0.722	-0.136	-0.429
207	222	26353	HSPB8	-0.220	-0.634	-0.427
116	223	150094	SNF1LK	0.268	-1.120	-0.426
604	224	55872	TOPK	-1.258	0.407	-0.426
334	225	7867	MAPKAPK3	-0.375	-0.470	-0.422
510	226	545	ATR	-0.257	-0.569	-0.413
332	227	79934	ADCK4	-0.225	-0.589	-0.407
255	228	22848	AAK1	-0.445	-0.369	-0.407
594	229	285220	EPHA6	-1.284	0.479	-0.402
335	230	93	ACVR2B	-0.479	-0.324	-0.402
7	231	65268	PRKWNK2	-0.027	-0.768	-0.398
343	232	5260	PHKG1	-0.500	-0.294	-0.397
685	233	5604	MAP2K1	-0.776	-0.007	-0.392
414	234	10595	ERN2	-0.695	-0.083	-0.389

185	235	10188	TNK2	0.048	-0.807	-0.380
516	236	57410	SCYL1	-1.162	0.407	-0.378
305	237	2710	GK	-0.538	-0.208	-0.373
118	238	9113	LATS1	0.138	-0.878	-0.370
432	239	5255	PHKA1	-0.831	0.096	-0.368
623	240	5128	PCTK2	-0.165	-0.569	-0.367
5	241	80347	COASY	-0.427	-0.303	-0.365
106	242	6787	NEK4	-0.227	-0.497	-0.362
271	243	2051	EPHB6	-0.486	-0.238	-0.362
702	244	2712	GK2	-0.117	-0.606	-0.361
572	245	1119	CHKA	-0.297	-0.418	-0.358
124	246	122481	AK7	-0.400	-0.312	-0.356
603	247	2965	GTF2H1	-0.754	0.047	-0.354
687	248	4914	NTRK1	-0.596	-0.108	-0.352
646	249	3611	ILK	-0.544	-0.155	-0.350
284	250	92	ACVR2	-0.815	0.135	-0.340
250	251	6046	BRD2	-0.386	-0.294	-0.340
143	252	65061	ALS2CR7	-0.270	-0.399	-0.335
413	253	29110	TBK1	-0.547	-0.116	-0.331
412	254	1432	MAPK14	-0.357	-0.306	-0.331
703	255	91419	KUB3	0.351	-1.013	-0.331
531	256	3551	IKBKB	-0.047	-0.606	-0.326
386	257	6732	SRPK1	-0.845	0.200	-0.322
539	258	9748	SLK	0.071	-0.710	-0.320
525	259	7016	TESK1	-0.960	0.342	-0.309
252	260	5606	MAP2K3	-0.398	-0.220	-0.309
663	261	5591	PRKDC	-0.367	-0.249	-0.308
383	262	5754	PTK7	-0.538	-0.050	-0.294
696	263	4356	MPP3	-0.334	-0.249	-0.291
352	264	83942	STK22D	-0.743	0.162	-0.291
369	265	8877	SPHK1	-0.715	0.135	-0.290
228	266	9863	AIP1	0.216	-0.792	-0.288
697	267	1018	CDK3	-0.507	-0.058	-0.282
261	268	8536	CAMK1	-0.413	-0.148	-0.281
204	269	10922	FASTK	0.195	-0.756	-0.281
85	270	29904	EEF2K	-0.241	-0.312	-0.277
643	271	51765	RP6-213H19.1	-0.172	-0.379	-0.275
440	272	1196	CLK2	-0.363	-0.178	-0.271
82	273	84451	KIAA1804	-0.436	-0.095	-0.265
555	274	6725	SRMS	-0.065	-0.458	-0.262

391	275	64710	NUCKS	-2.114	1.595	-0.260
417	276	122011	CSNK1A1L	-0.450	-0.059	-0.254
254	277	10518	CIB2	-0.363	-0.136	-0.250
415	278	5584	PRKCI	-0.470	-0.026	-0.248
673	279	5579	PRKCB1	-0.967	0.479	-0.244
342	280	1969	EPHA2	-0.391	-0.092	-0.241
582	281	4117	MAK	-0.422	-0.058	-0.240
272	282	4139	MARK1	-0.641	0.162	-0.239
249	283	3717	JAK2	-0.500	0.022	-0.239
489	284	8621	CDC2L5	-0.275	-0.202	-0.239
6	285	140469	MYO3B	-0.139	-0.330	-0.234
187	286	558	AXL	0.100	-0.545	-0.222
581	287	4831	NME2	-0.139	-0.292	-0.216
173	288	699	BUB1	-0.207	-0.187	-0.197
291	289	7046	TGFBR1	-0.561	0.174	-0.194
452	290	5832	PYCS	-0.584	0.200	-0.192
402	291	9414	TJP2	-0.665	0.287	-0.189
27	292	51265	CDKL3	0.359	-0.735	-0.188
546	293	1399	CRKL	-0.533	0.158	-0.187
506	294	2041	EPHA1	-0.993	0.627	-0.183
263	295	167359	MGC42105	-0.386	0.022	-0.182
309	296	4920	ROR2	-0.184	-0.178	-0.181
678	297	5577	PRKAR2B	-0.691	0.342	-0.175
666	298	85443	KIAA1765	-1.052	0.706	-0.173
382	299	26576	STK23	-0.525	0.188	-0.168
199	300	91754	NEK9	0.334	-0.655	-0.161
504	301	11329	STK38	-0.724	0.407	-0.159
660	302	2987	GUK1	-0.065	-0.249	-0.157
303	303	8566	PDXK	-0.275	-0.038	-0.156
95	304	5681	PSKH1	0.431	-0.735	-0.152
113	305	85366	MYLK2	-0.361	0.066	-0.147
464	306	5588	PRKCQ	-0.177	-0.116	-0.146
321	307	4296	MAP3K11	0.025	-0.276	-0.126
379	308	5616	PRKY	-0.423	0.174	-0.124
583	309	5293	PIK3CD	-0.165	-0.058	-0.111
115	310	57551	KIAA1361	-0.277	0.066	-0.105
258	311	130399	ACVR1C	-0.466	0.257	-0.104
560	312	9833	MELK	0.292	-0.497	-0.103
463	313	1032	CDKN2D	-0.391	0.188	-0.101
642	314	9942	XYLB	0.137	-0.335	-0.099

595	315	2984	GUCY2C	-0.824	0.627	-0.098
408	316	80216	LAK	-0.198	0.010	-0.094
315	317	6198	RPS6KB1	-0.402	0.215	-0.093
420	318	56155	TEX14	-0.177	-0.002	-0.090
591	319	5313	PKLR	-0.172	-0.007	-0.090
593	320	7443	VRK1	-0.393	0.216	-0.088
54	321	1164	CKS2	-0.302	0.156	-0.073
497	322	2444	FRK	-0.073	-0.058	-0.065
607	323	1147	CHUK	-0.161	0.047	-0.057
692	324	9874	TLK1	-0.452	0.342	-0.055
543	325	5105	PCK1	0.225	-0.335	-0.055
528	326	5210	PFKFB4	-0.209	0.101	-0.054
107	327	53944	CSNK1G1	0.431	-0.530	-0.049
117	328	5756	PTK9	-0.416	0.323	-0.047
676	329	166614	MGC45428	-0.500	0.407	-0.046
142	330	51086	TNNI3K	0.247	-0.339	-0.046
532	331	9175	MAP3K13	-0.367	0.277	-0.045
105	332	6714	SRC	-0.002	-0.083	-0.043
373	333	4919	ROR1	-0.593	0.510	-0.041
548	334	282974	STK32C	0.255	-0.335	-0.040
191	335	3718	JAK3	0.431	-0.506	-0.037
397	336	659	BMPR2	-0.311	0.242	-0.035
585	337	676	BRDT	-0.680	0.627	-0.027
439	338	6733	SRPK2	-0.282	0.230	-0.026
411	339	124923	FLJ25006	-0.114	0.069	-0.022
429	340	83931	MGC4796	-0.491	0.460	-0.015
176	341	5298	PIK4CB	0.186	-0.217	-0.015
198	342	3480	IGF1R	0.109	-0.136	-0.014
349	343	613	BCR	-0.357	0.332	-0.013
63	344	55750	MULK	0.418	-0.440	-0.011
614	345	9451	EIF2AK3	-0.297	0.277	-0.010
401	346	11011	TLK2	-0.318	0.302	-0.008
574	347	6195	RPS6KA1	0.450	-0.458	-0.004
317	348	146057	TTBK2	-0.352	0.346	-0.003
48	349	23683	PRKCN	0.302	-0.265	0.019
336	350	1956	EGFR	-0.123	0.162	0.019
672	351	2049	EPHB3	-0.584	0.627	0.021
658	352	805	CALM2	0.056	-0.007	0.024
598	353	5156	PDGFRA	-0.165	0.216	0.026
299	354	64768	C9ORF12	-0.509	0.564	0.028

339	355	4921	DDR2	0.150	-0.092	0.029
337	356	6098	ROS1	-0.286	0.346	0.030
259	357	91	ACVR1B	0.043	0.022	0.032
333	358	27102	HRI	0.043	0.034	0.038
533	359	57118	CAMK1D	-0.330	0.407	0.038
542	360	5211	PFKL	0.369	-0.292	0.039
292	361	8941	CDK5R2	-0.218	0.302	0.042
617	362	5287	PIK3C2B	-0.316	0.407	0.046
522	363	11035	RIPK3	-0.124	0.216	0.046
537	364	6789	STK4	-0.249	0.342	0.046
47	365	9448	MAP4K4	0.302	-0.208	0.047
602	366	202374	STK32A	-0.452	0.551	0.050
26	367	50488	MINK	0.166	-0.062	0.052
655	368	1017	CDK2	0.163	-0.058	0.053
89	369	8986	RPS6KA4	0.256	-0.136	0.060
197	370	55224	FLJ10761	0.032	0.090	0.061
330	371	5570	PKIB	0.034	0.096	0.065
474	372	30849	PIK3R4	-0.093	0.230	0.068
597	373	51231	VRK3	-0.397	0.551	0.077
112	374	5164	PDK2	0.057	0.105	0.081
523	375	54986	ULK4	-0.463	0.627	0.082
693	376	5213	PFKM	-0.378	0.551	0.086
695	377	3707	ITPKB	0.078	0.101	0.089
670	378	1263	PLK3	0.148	0.047	0.097
418	379	8394	PIP5K1A	-0.136	0.332	0.098
24	380	27	ABL2	0.084	0.117	0.100
632	381	8550	MAPKAPK5	0.538	-0.335	0.102
683	382	1739	DLG1	-0.492	0.706	0.107
612	383	11200	CHEK2	-0.179	0.407	0.114
56	384	5598	MAPK7	0.511	-0.273	0.119
310	385	79837	PIP5K2C	-0.232	0.478	0.123
534	386	11213	IRAK3	-0.305	0.551	0.123
512	387	3791	KDR	-0.934	1.185	0.126
205	388	5593	PRKG2	0.443	-0.178	0.132
535	389	4342	MOS	-0.283	0.551	0.134
38	390	83983	SSTK	0.075	0.194	0.135
37	391	5297	PIK4CA	0.345	-0.071	0.137
290	392	5599	MAPK8	0.086	0.188	0.137
398	393	340371	NRBP2	0.113	0.174	0.143
538	394	9223	BAIAP1	0.303	-0.007	0.148

238	395	7525	YES1	0.690	-0.366	0.162
318	396	8476	CDC42BPA	0.141	0.188	0.165
653	397	5564	PRKAB1	0.284	0.047	0.166
300	398	5753	PTK6	-0.232	0.564	0.166
133	399	54963	URKL1	0.177	0.156	0.166
476	400	2048	EPHB2	0.009	0.332	0.170
127	401	9950	GOLGA5	0.595	-0.247	0.174
713	402	8859	STK19	0.815	-0.458	0.178
588	403	1859	DYRK1A	-0.264	0.627	0.181
184	404	5601	MAPK9	0.381	-0.017	0.182
579	405	390226	LOC390226	0.789	-0.418	0.185
351	406	93627	MGC16169	0.043	0.332	0.187
338	407	3101	HK3	0.034	0.346	0.190
360	408	55300	PI4K2B	0.034	0.346	0.190
251	409	1031	CDKN2C	-0.130	0.510	0.190
712	410	60385	TSKS	0.630	-0.249	0.191
656	411	5605	MAP2K2	0.225	0.158	0.192
395	412	9943	OSR1	-0.329	0.719	0.195
578	413	5062	PAK2	0.292	0.101	0.196
700	414	2043	EPHA4	0.410	-0.007	0.201
576	415	660	BMX	0.130	0.277	0.204
316	416	55312	RFK	-0.211	0.621	0.205
625	417	147746	HIPK4	0.310	0.101	0.205
43	418	3984	LIMK1	0.334	0.078	0.206
536	419	8396	PIP5K2B	-0.139	0.551	0.206
186	420	149420	PDIK1L	0.456	-0.038	0.209
331	421	1020	CDK5	0.025	0.394	0.209
688	422	4833	NME4	-0.194	0.627	0.216
175	423	5587	PRKCM	0.216	0.236	0.226
498	424	5291	PIK3CB	0.181	0.277	0.229
31	425	983	CDC2	0.538	-0.071	0.234
465	426	51422	PRKAG2	-0.184	0.659	0.238
138	427	3815	KIT	0.795	-0.312	0.241
518	428	83931	MGC4796	0.016	0.479	0.247
181	429	1454	CSNK1E	0.497	0.019	0.258
39	430	9344	TAO1	0.302	0.221	0.262
577	431	57396	CLK4	0.310	0.216	0.263
615	432	5289	PIK3C3	-0.172	0.706	0.267
137	433	55450	CAMKIINALPHA	0.674	-0.136	0.269
104	434	8382	NME5	0.302	0.236	0.269

467	435	53834	FGFRL1	-0.007	0.546	0.270
662	436	8491	MAP4K3	-0.010	0.551	0.270
634	437	5208	PFKFB2	0.653	-0.108	0.272
164	438	1608	DGKG	0.524	0.031	0.277
556	439	5871	MAP4K2	0.399	0.158	0.278
55	440	5261	PHKG2	0.279	0.278	0.279
64	441	2870	GRK6	0.497	0.066	0.282
657	442	5602	MAPK10	0.016	0.551	0.283
505	443	10654	PMVK	-0.607	1.185	0.289
136	444	10645	CAMKK2	0.674	-0.095	0.290
407	445	5607	MAP2K5	-0.039	0.621	0.291
622	446	11184	MAP4K1	0.104	0.479	0.292
165	447	23031	MAST3	0.443	0.141	0.292
135	448	3000	GUCY2D	0.690	-0.104	0.293
545	449	2264	FGFR4	0.255	0.342	0.299
694	450	5563	PRKAA2	-0.179	0.793	0.307
370	451	10926	ASK	0.188	0.427	0.308
57	452	131096	KCNH8	0.931	-0.312	0.309
53	453	1456	CSNK1G3	0.157	0.463	0.310
167	454	10295	BCKDK	0.511	0.129	0.320
421	455	9261	MAPKAPK2	-0.032	0.677	0.323
33	456	120892	LRRK2	0.177	0.481	0.329
381	457	7535	ZAP70	0.077	0.582	0.329
121	458	55781	RIOK2	0.381	0.278	0.330
509	459	5580	PRKCD	-0.216	0.883	0.333
647	460	85481	PSKH2	0.262	0.407	0.335
214	461	1326	MAP3K8	0.406	0.263	0.335
301	462	5058	PAK1	-0.157	0.826	0.335
449	463	1026	CDKN1A	0.018	0.659	0.339
103	464	5218	PFTK1	0.227	0.463	0.345
571	465	51727	UMP-CMPK	0.605	0.101	0.353
215	466	1024	CDK8	0.776	-0.062	0.357
302	467	6259	RYK	0.095	0.639	0.367
601	468	5286	PIK3C2A	0.038	0.706	0.372
394	469	79834	KIAA2002	0.050	0.698	0.374
163	470	23533	P101-PI3K	0.334	0.415	0.374
459	471	23139	MAST2	0.338	0.427	0.383
166	472	1944	EFNA3	0.256	0.513	0.385
573	473	65266	WNK4	0.505	0.277	0.391
640	474	55728	N4BP2	0.737	0.047	0.392

511	475	815	CAMK2A	0.630	0.158	0.394
520	476	10454	MAP3K7IP1	-0.080	0.883	0.401
58	477	817	CAMK2D	0.849	-0.038	0.406
182	478	5970	RELA	0.758	0.066	0.412
192	479	83942	STK22D	0.642	0.183	0.412
311	480	90	ACVR1	0.000	0.826	0.413
183	481	23552	CCRK	0.497	0.352	0.425
613	482	3716	JAK1	-0.010	0.883	0.436
211	483	6197	RPS6KA3	0.758	0.117	0.438
641	484	4750	NEK1	0.664	0.216	0.440
675	485	2050	EPHB4	-0.305	1.185	0.440
123	486	5157	PDGFRL	0.674	0.209	0.442
547	487	9252	RPS6KA5	0.351	0.551	0.451
134	488	4293	MAP3K9	0.581	0.323	0.452
392	489	11113	CIT	0.197	0.719	0.458
42	490	2869	GRK5	0.722	0.221	0.472
174	491	127933	UHMK1	0.595	0.367	0.481
312	492	5567	PRKACB	0.270	0.698	0.484
580	493	1452	CSNK1A1	0.432	0.551	0.491
314	494	4145	MATK	-0.048	1.044	0.498
450	495	80025	PANK2	0.034	0.966	0.500
494	496	5603	MAPK13	0.303	0.706	0.504
479	497	5979	RET	0.350	0.659	0.504
627	498	4233	MET	0.737	0.277	0.507
132	499	818	CAMK2G	0.795	0.221	0.508
41	500	53632	PRKAG3	0.345	0.680	0.513
111	501	84446	KIAA1811	0.795	0.236	0.515
396	502	5891	RAGE	0.229	0.805	0.517
168	503	3706	ITPKA	0.256	0.782	0.519
69	504	8576	STK16	0.849	0.194	0.522
15	505	84930	MASTL	0.538	0.531	0.535
177	506	1742	DLG4	0.795	0.278	0.536
209	507	8445	DYRK2	0.813	0.278	0.545
502	508	9625	AATK	-0.334	1.427	0.546
234	509	10733	PLK4	0.994	0.117	0.556
492	510	112858	TP53RK	1.072	0.047	0.559
28	511	9020	MAP3K14	0.951	0.168	0.559
587	512	10201	NME6	-0.179	1.301	0.561
189	513	8737	RIPK1	0.484	0.641	0.562
203	514	1612	DAPK1	0.931	0.194	0.563

108	515	83732	RIOK1	0.974	0.156	0.565
213	516	57143	ADCK1	1.042	0.090	0.566
66	517	65975	STK33	1.090	0.042	0.566
690	518	23178	PASK	0.726	0.407	0.567
16	519	10221	TRIB1	0.951	0.194	0.573
410	520	79705	LRRK1	0.499	0.659	0.579
52	521	53354	PANK1	0.910	0.251	0.581
172	522	4355	MPP2	0.931	0.236	0.584
470	523	9024	STK29	0.409	0.761	0.585
637	524	260425	MAGI-3	0.549	0.627	0.588
659	525	3795	KHK	1.234	-0.058	0.588
178	526	695	BTK	0.674	0.513	0.594
680	527	269	AMHR2	0.572	0.627	0.599
471	528	4916	NTRK3	0.281	0.919	0.600
32	529	11040	PIM2	0.740	0.463	0.601
128	530	8428	STK24	0.931	0.278	0.604
145	531	1163	CKS1B	1.194	0.019	0.606
621	532	30811	HUNK	0.538	0.706	0.622
188	533	54101	ANKRD3	0.642	0.603	0.623
212	534	1945	EFNA4	1.090	0.168	0.629
409	535	1022	CDK7	0.209	1.071	0.640
715	536	2584	GALK1	0.881	0.407	0.644
236	537	7301	TYRO3	0.890	0.400	0.645
313	538	27347	STK39	0.170	1.127	0.649
521	539	5610	PRKR	0.321	0.976	0.649
202	540	5305	PIP5K2A	0.931	0.367	0.649
49	541	84630	TTBK1	0.890	0.415	0.652
679	542	5106	PCK2	0.016	1.301	0.658
393	543	344387	CDKL4	0.327	0.993	0.660
67	544	57538	MIDORI	1.194	0.129	0.662
237	545	5595	MAPK3	1.342	-0.017	0.662
708	546	4486	MST1R	1.433	-0.108	0.662
701	547	9201	DCAMKL1	1.234	0.101	0.667
217	548	5288	PIK3C2G	1.090	0.263	0.676
13	549	9061	PAPSS1	1.065	0.293	0.679
169	550	10783	NEK6	0.511	0.871	0.691
260	551	8895	CPNE3	0.043	1.339	0.691
225	552	57172	CAMK1G	1.278	0.105	0.692
23	553	132	ADK	0.627	0.761	0.694
529	554	9088	PKMYT1	0.310	1.077	0.694

146	555	55589	BMP2K	1.140	0.251	0.695
218	556	1019	CDK4	1.167	0.236	0.702
478	557	1741	DLG3	-0.071	1.479	0.704
711	558	65125	PRKWNK1	1.010	0.407	0.708
216	559	8569	MKNK1	1.065	0.352	0.709
65	560	658	BMPR1B	1.140	0.278	0.709
706	561	2986	GUCY2F	0.538	0.883	0.711
19	562	3098	HK1	0.890	0.531	0.711
17	563	8019	BRD3	1.042	0.382	0.712
219	564	5292	PIM1	1.017	0.415	0.716
144	565	3055	HCK	1.222	0.221	0.721
558	566	5163	PDK1	0.965	0.479	0.722
141	567	2872	MKNK2	0.706	0.740	0.723
14	568	5596	MAPK4	0.722	0.740	0.731
18	569	197259	FLJ34389	0.974	0.513	0.744
109	570	207	AKT1	0.870	0.641	0.756
119	571	5214	PFKP	0.813	0.701	0.757
628	572	2534	FYN	1.120	0.407	0.764
93	573	2044	EPHA5	0.849	0.680	0.765
40	574	4832	NME3	0.795	0.740	0.767
9	575	2241	FER	0.256	1.297	0.777
96	576	6795	AURKC	1.310	0.263	0.787
611	577	114783	LMTK3	0.273	1.301	0.787
618	578	6446	SGK	0.505	1.077	0.791
495	579	28951	TRIB2	0.881	0.706	0.793
94	580	91807	MLCK	0.994	0.624	0.809
468	581	4830	NME1	0.692	0.942	0.817
224	582	138474	TAF1L	1.478	0.168	0.823
569	583	3482	IGF2R	1.827	-0.155	0.836
101	584	9060	PAPSS2	1.478	0.194	0.836
380	585	4214	MAP3K1	0.629	1.044	0.836
475	586	2645	GCK	0.338	1.339	0.839
319	587	83903	GSG2	0.742	0.942	0.842
681	588	63904	DUSP21	0.505	1.185	0.845
102	589	6199	RPS6KB2	0.831	0.871	0.851
131	590	8317	CDC7	0.994	0.719	0.857
270	591	5609	MAP2K7	0.188	1.556	0.872
460	592	8851	CDK5R1	0.197	1.556	0.877
540	593	1028	CDKN1C	0.965	0.793	0.879
147	594	5257	PHKB	1.278	0.481	0.879

21	595	55437	ALS2CR2	0.951	0.826	0.889
221	596	7786	MAP3K12	1.115	0.680	0.898
552	597	23412	COMMD3	1.532	0.277	0.905
227	598	6793	STK10	1.278	0.549	0.914
541	599	23396	PIP5K1C	1.617	0.216	0.917
389	600	10461	MERTK	0.540	1.306	0.923
99	601	140803	TRPM6	1.249	0.603	0.926
29	602	6885	MAP3K7	0.870	0.987	0.928
129	603	1607	DGKB	1.140	0.719	0.929
92	604	80271	ITPKC	1.042	0.826	0.934
553	605	7084	TK2	1.532	0.342	0.937
11	606	94	ACVRL1	0.890	0.987	0.939
635	607	57787	MARK4	0.815	1.077	0.946
231	608	79672	FN3KRP	0.910	0.987	0.949
500	609	8814	CDKL1	0.829	1.077	0.953
239	610	89882	NYD-SP25	0.994	0.916	0.955
419	611	9263	STK17A	0.397	1.518	0.957
12	612	4067	LYN	1.042	0.892	0.967
200	613	84197	FLJ23356	0.813	1.148	0.980
709	614	204	AK2	1.415	0.551	0.983
609	615	283629	C14ORF20	1.577	0.407	0.992
631	616	4751	NEK2	1.433	0.551	0.992
590	617	51347	JIK	1.120	0.883	1.001
651	618	8999	CDKL2	1.120	0.883	1.001
390	619	64781	CERK	0.761	1.244	1.002
235	620	10114	HIPK3	1.408	0.603	1.005
179	621	389840	MAP3K15	1.065	0.963	1.014
570	622	7049	TGFBR3	1.323	0.706	1.014
469	623	8711	TNK1	0.645	1.407	1.026
620	624	54899	PXK	1.102	0.976	1.039
636	625	2047	EPHB1	1.396	0.706	1.051
75	626	1455	CSNK1G2	1.342	0.761	1.051
499	627	3757	KCNH2	1.779	0.342	1.061
340	628	23604	DAPK2	0.815	1.339	1.077
50	629	6093	ROCK1	0.994	1.175	1.085
161	630	10746	MAP3K2	1.278	0.892	1.085
691	631	3364	HUS1	0.995	1.185	1.090
490	632	238	ALK	1.555	0.627	1.091
59	633	11183	MAP4K5	1.442	0.740	1.091
638	634	83983	SSTK	1.779	0.407	1.093

477	635	23678	SGKL	0.434	1.768	1.101
399	636	6416	MAP2K4	0.692	1.518	1.105
140	637	8767	RIPK2	1.342	0.871	1.106
139	638	5585	PRKCL1	1.442	0.782	1.112
557	639	84446	KIAA1811	1.359	0.883	1.121
156	640	10110	SGK2	1.553	0.701	1.127
90	641	801	CALM1	0.706	1.577	1.142
91	642	152110	FLJ32685	1.194	1.092	1.143
130	643	10494	STK25	1.553	0.740	1.146
68	644	6041	RNASEL	1.594	0.719	1.156
699	645	118672	C10ORF89	1.341	0.976	1.159
201	646	2322	FLT3	1.249	1.092	1.170
171	647	139189	DGKK	1.140	1.205	1.172
98	648	203	AK1	1.042	1.327	1.185
350	649	1633	DCK	1.067	1.339	1.203
30	650	1152	CKB	1.017	1.395	1.206
51	651	7010	TEK	0.951	1.464	1.208
155	652	5129	PCTK3	1.222	1.205	1.213
650	653	5578	PRKCA	1.135	1.301	1.218
157	654	8798	DYRK4	1.726	0.719	1.222
170	655	640	BLK	1.017	1.428	1.223
22	656	23729	CARKL	1.278	1.175	1.227
226	657	55561	HSMDPKIN	1.594	0.871	1.232
222	658	81629	STK22C	1.140	1.360	1.250
180	659	2931	GSK3A	1.042	1.464	1.253
77	660	23043	TNIK	1.442	1.092	1.267
661	661	1158	CKM	1.120	1.427	1.273
162	662	26524	LATS2	1.514	1.038	1.276
62	663	5256	PHKA2	1.249	1.327	1.288
554	664	1457	CSNK2A1	1.323	1.301	1.312
233	665	23387	KIAA0999	1.442	1.205	1.323
710	666	140609	NEK7	1.577	1.077	1.327
154	667	51135	IRAK4	1.514	1.148	1.331
148	668	7204	TRIO	1.553	1.118	1.336
669	669	50808	AK3L1	1.120	1.564	1.342
649	670	8408	ULK1	1.323	1.427	1.375
210	671	5586	PRKCL2	1.408	1.360	1.384
223	672	55361	PI4KII	1.374	1.395	1.385
549	673	25865	PRKD2	1.216	1.564	1.390
320	674	204851	HIPK1	0.761	2.024	1.392

158	675	55229	PANK4	1.408	1.428	1.418
149	676	55500	ETNK1	1.594	1.264	1.429
61	677	472	ATM	1.115	1.744	1.429
70	678	6011	GRK1	1.773	1.092	1.432
491	679	10290	SPEG	1.709	1.185	1.447
76	680	2011	MARK2	1.635	1.264	1.450
120	681	5592	PRKG1	1.726	1.175	1.450
190	682	9578	CDC42BPB	1.478	1.464	1.471
630	683	22983	SAST	2.066	0.883	1.474
229	684	25	ABL1	1.594	1.360	1.477
519	685	55351	STK32B	1.577	1.427	1.502
633	686	203447	NRK	1.304	1.711	1.508
60	687	29941	PKN3	1.932	1.092	1.512
220	688	1609	DGKQ	1.514	1.577	1.546
230	689	56924	PAK6	1.773	1.360	1.567
639	690	8517	IKBKG	2.350	0.793	1.571
74	691	10769	PLK2	1.478	1.699	1.589
20	692	79896	THNSL1	1.310	1.884	1.597
110	693	54861	SNRK	1.635	1.658	1.646
152	694	5294	PIK3CG	1.442	1.884	1.663
79	695	5315	PKM2	1.994	1.395	1.695
153	696	2261	FGFR3	1.514	1.884	1.699
71	697	5987	RFP	0.974	2.436	1.705
80	698	6792	CDKL5	1.994	1.428	1.711
232	699	208	AKT2	1.635	1.837	1.736
100	700	1195	CLK1	1.932	1.699	1.816
73	701	341676	NEK5	1.878	1.789	1.833
151	702	83440	ADP-GK	1.514	2.161	1.838
240	703	81602	CDADC1	2.125	1.577	1.851
159	704	5568	PRKACG	2.277	1.500	1.889
619	705	1198	CLK3	2.217	1.564	1.890
78	706	808	CALM3	2.125	1.658	1.892
550	707	84254	CAMKK1	2.250	1.564	1.907
559	708	253430	IPMK	2.571	1.427	1.999
599	709	780	DDR1	2.611	1.427	2.019
551	710	92335	LYK5	2.493	1.564	2.028
150	711	225689	ERK8	2.677	1.539	2.108
72	712	138429	PIP5KL1	1.726	2.597	2.161
629	713	25989	DKFZP434C131	2.696	1.877	2.286
160	714	2321	FLT1	2.277	2.361	2.319

589	715	814	CAMK4	2.387	2.511	2.449
